# 1,4-Diazo­niabicyclo­[2.2.2]octane bis­(2,4,6-trinitro­phenolate)

**DOI:** 10.1107/S1600536810021021

**Published:** 2010-06-05

**Authors:** Weiwei SiMa

**Affiliations:** a Ordered Matter Science Research Center, Southeast University, Nanjing 210096, People’s Republic of China

## Abstract

In the title compound, C_6_H_14_N_2_^2+^·2C_6_H_2_N_3_O_7_^−^, the cation possesses crystallographically imposed twofold rotation symmetry. In the crystal structure, the cation and anions are linked into a trimeric aggregate by inter­molecular N—H⋯O hydrogen bonds. The trimeric units are further connected by π–π inter­actions [centroid–centroid distances = 3.507 (2)–3.660 (3) Å], forming layers parallel to the *bc* plane.

## Related literature

For a discussion on hydrogen bonding in in the title crystal, see: Kumai *et al.* (2007 ▶); Horiuchi *et al.* (2005 ▶). For related structures, see: Dabros *et al.* (2007 ▶); Jin *et al.* (2004 ▶); Glidewell *et al.* (1999 ▶); Chen *et al.* (2009 ▶).
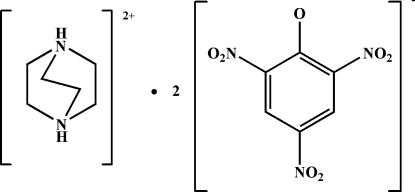


## Experimental

### 

#### Crystal data

C_6_H_14_N_2_^2+^·2C_6_H_2_N_3_O_7_^−^
*M* *_r_* = 570.40
Monoclinic, 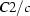

*a* = 15.3808 (11) Å
*b* = 7.1520 (5) Å
*c* = 25.3527 (14) Å
β = 125.496 (2)°
*V* = 2270.6 (3) Å^3^
*Z* = 4
Mo *K*α radiation
μ = 0.15 mm^−1^
*T* = 93 K
0.1 × 0.1 × 0.1 mm

#### Data collection

Rigaku SCXmini diffractometer
Absorption correction: multi-scan (*CrystalClear*; Rigaku, 2005 ▶) *T*_min_ = 0.857, *T*_max_ = 1.000
10700 measured reflections
2590 independent reflections
2218 reflections with *I* > 2σ(*I*)
*R*_int_ = 0.028

#### Refinement

*R*[*F* ^2^ > 2σ(*F* ^2^)] = 0.039
*wR*(*F* ^2^) = 0.098
*S* = 1.07
2590 reflections
181 parameters
H-atom parameters constrained
Δρ_max_ = 0.54 e Å^−3^
Δρ_min_ = −0.59 e Å^−3^

### 

Data collection: *CrystalClear* (Rigaku, 2005 ▶); cell refinement: *CrystalClear* (Rigaku, 2005 ▶); data reduction: *CrystalClear* (Rigaku, 2005 ▶); program(s) used to solve structure: *SHELXS97* (Sheldrick, 2008 ▶); program(s) used to refine structure: *SHELXL97* (Sheldrick, 2008 ▶); molecular graphics: *SHELXTL* (Sheldrick, 2008 ▶); software used to prepare material for publication: *PRPKAPPA* (Ferguson, 1999 ▶).

## Supplementary Material

Crystal structure: contains datablocks I, global. DOI: 10.1107/S1600536810021021/rz2442sup1.cif

Structure factors: contains datablocks I. DOI: 10.1107/S1600536810021021/rz2442Isup2.hkl

Additional supplementary materials:  crystallographic information; 3D view; checkCIF report

## Figures and Tables

**Table 1:** Hydrogen-bond geometry (Å, °)

*D*—H⋯*A*	*D*—H	H⋯*A*	*D*⋯*A*	*D*—H⋯*A*
N4—H4*A*⋯O1	0.93	1.69	2.589 (2)	161
N4—H4*A*⋯O2	0.93	2.42	2.954 (2)	117

**Fig. 1.**
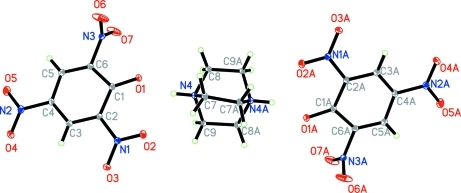
The molecular structure of the title compound, with the atomic numbering scheme. Displacement ellipsoids are drawn at the 30% probability level. Atoms labelled with suffix A are generated by the symmetry operation (-x, y, 0.5-z).

**Fig. 2.**
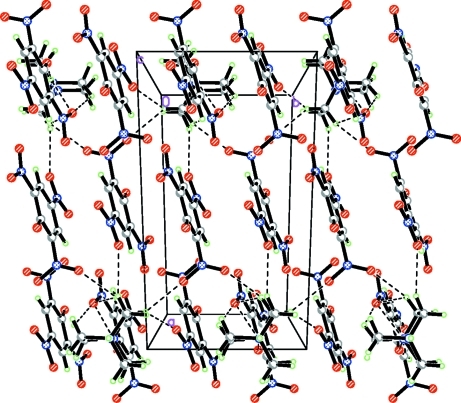
Crystal packing of the title compound viewed along the c axis. Dashed lines indicate hydrogen bonds.

## supplementary crystallographic information

### Comment

The co-crystals of 1,4-diazabicyclo[2.2.2]octane (DABCO) and phenols are typically characterized by the presence of N—H···O or O—H···N hydrogen-bonded adducts (Kumai *et al.* (2007); Horiuchi *et al.*, 2005). Many of this type of co-crystals have been designed by employing crystal-engineering strategies, and their structures have been studied extensively (Dabros *et al.*, 2007; Jin *et al.*, 2004; Glidewell *et al.*, 1999). As a continuation of a study of phase transitions in hydrogen-bonded co-crystalline compounds between phenols and tertiary amines as N–H···O-type systems (Chen *et al.*, 2009), the crystal structure of the 1:2 co-crystal of DABCO and 2,4,6-trinitrophenol obtained by a single-crystal X-ray analysis is reported herein. The compound shows no dielectric irregularity in the temperature range of 93–373K.

The title compound (Fig. 1) was obtained from the reaction of 1,4-diazabicyclo[2.2.2]octane and 2,4,6-trinitrophenol. The cation has crystallographically imposed twofold rotation symmetry. The two protonated N atoms in the cation are almost equivalent with very close C–N bond lengths [1.4930 (19) to 1.4952 (18) Å] and C–N–C angles [109.79 (11)° to 110.72 (11)°]. Within the benzene ring of the 2,4,6-trinitrophenol anion, the C–C–C bond angles of the three nitro-connected C atoms are in the range 121.92 (13)–126.76 (13)°, and are a little larger than the remaining three C–C–C bond angles. In the crystal structure (Fig. 2), cation and anions are linked into a trimeric aggregate by intermolecular N—H···O hydrogen bonds (Table 1). The trimeric units are further connected by π–π interactions (centroid-to-centroid distance = 3.507 (2)–3.660 (3) Å) to form layers parallel to the *bc* plane.

### Experimental

1,4-Diazabicyclo[2.2.2]octane (DABCO) (2.5 mmol) was dissolved in ethanol (10 ml).The clear solution obtained was added to a solution of 2,4,6-trinitrophenol(5 mmol) in ethanol (20 ml). The formed precipitate was then filtered and the obtained yellow solid was redissolved in DMF (15 ml). Yellow co-crystals of the title compound suitable for X-ray diffraction analysis were obtained by slow evaporation of the mixture at room temperature after 7 days.

### Refinement

All the H atoms were calculated geometrically and were allowed to ride, with C—H = 0.95-0.99 Å, N—H = 0.93 Å, and with *U*_iso_(H) = 1.2*U*_eq_(C, N).

### Crystal data

**Table:**

C_6_H_14_N_2_^2+^·2C_6_H_2_N_3_O_7_^−^	*F*(000) = 1176
*M**_r_* = 570.40	*D*_x_ = 1.669 Mg m^−^^3^
Monoclinic, *C*2/*c*	Mo *K*α radiation, λ = 0.71075 Å
Hall symbol: -C 2yc	Cell parameters from 4042 reflections
*a* = 15.3808 (11) Å	θ = 3.5–27.6°
*b* = 7.1520 (5) Å	µ = 0.15 mm^−^^1^
*c* = 25.3527 (14) Å	*T* = 93 K
β = 125.496 (2)°	Prism, yellow
*V* = 2270.6 (3) Å^3^	0.1 × 0.1 × 0.1 mm
*Z* = 4	

### Data collection

**Table:**

Rigaku SCXmini diffractometer	2590 independent reflections
Radiation source: fine-focus sealed tube	2218 reflections with *I* > 2σ(*I*)
graphite	*R*_int_ = 0.028
Detector resolution: 13.6612 pixels mm^-1^	θ_max_ = 27.5°, θ_min_ = 3.1°
ω scans	*h* = −19→18
Absorption correction: multi-scan (*CrystalClear*; Rigaku, 2005)	*k* = −9→9
*T*_min_ = 0.857, *T*_max_ = 1.000	*l* = −32→32
10700 measured reflections	

### Refinement

**Table:**

Refinement on *F*^2^	Primary atom site location: structure-invariant direct methods
Least-squares matrix: full	Secondary atom site location: difference Fourier map
*R*[*F*^2^ > 2σ(*F*^2^)] = 0.039	Hydrogen site location: inferred from neighbouring sites
*wR*(*F*^2^) = 0.098	H-atom parameters constrained
*S* = 1.07	*w* = 1/[σ^2^(*F*_o_^2^) + (0.0406*P*)^2^ + 3.4753*P*] where *P* = (*F*_o_^2^ + 2*F*_c_^2^)/3
2590 reflections	(Δ/σ)_max_ < 0.001
181 parameters	Δρ_max_ = 0.54 e Å^−^^3^
0 restraints	Δρ_min_ = −0.59 e Å^−^^3^

### Special details

**Table:**

Geometry. All esds (except the esd in the dihedral angle between two l.s. planes) are estimated using the full covariance matrix. The cell esds are taken into account individually in the estimation of esds in distances, angles and torsion angles; correlations between esds in cell parameters are only used when they are defined by crystal symmetry. An approximate (isotropic) treatment of cell esds is used for estimating esds involving l.s. planes.
Refinement. Refinement of *F*^2^ against ALL reflections. The weighted *R*-factor wR and goodness of fit *S* are based on *F*^2^, conventional *R*-factors *R* are based on *F*, with *F* set to zero for negative *F*^2^. The threshold expression of *F*^2^ > σ(*F*^2^) is used only for calculating *R*-factors(gt) etc. and is not relevant to the choice of reflections for refinement. *R*-factors based on *F*^2^ are statistically about twice as large as those based on *F*, and *R*- factors based on ALL data will be even larger.

### Fractional atomic coordinates and isotropic or equivalent isotropic displacement parameters (Å^2^)

**Table:**

	*x*	*y*	*z*	*U*_iso_*/*U*_eq_	
O1	0.06908 (8)	0.27695 (17)	0.12769 (5)	0.0196 (2)	
O2	−0.12313 (9)	0.44433 (16)	0.07242 (5)	0.0189 (2)	
O3	−0.23908 (8)	0.41893 (16)	−0.03110 (5)	0.0192 (2)	
O4	−0.13536 (9)	0.27577 (18)	−0.16941 (5)	0.0248 (3)	
O5	0.01746 (9)	0.14572 (17)	−0.13108 (5)	0.0241 (3)	
O6	0.28037 (11)	0.2978 (2)	0.13599 (8)	0.0563 (5)	
O7	0.23717 (10)	0.01540 (18)	0.13396 (7)	0.0404 (4)	
N1	−0.14821 (10)	0.39809 (17)	0.01833 (6)	0.0135 (3)	
N2	−0.04785 (10)	0.21799 (18)	−0.12408 (6)	0.0167 (3)	
N3	0.21729 (10)	0.17069 (18)	0.11151 (6)	0.0156 (3)	
N4	0.00848 (9)	0.28214 (18)	0.20398 (6)	0.0136 (3)	
H4A	0.0147	0.2825	0.1696	0.016*	
C1	0.03700 (11)	0.2720 (2)	0.06954 (7)	0.0135 (3)	
C2	−0.06664 (11)	0.3209 (2)	0.01256 (7)	0.0129 (3)	
C3	−0.09268 (11)	0.30461 (19)	−0.04961 (7)	0.0133 (3)	
H3A	−0.1616	0.3403	−0.0859	0.016*	
C4	−0.01856 (12)	0.2367 (2)	−0.05864 (7)	0.0143 (3)	
C5	0.08530 (12)	0.1875 (2)	−0.00629 (7)	0.0141 (3)	
H5A	0.1364	0.1409	−0.0126	0.017*	
C6	0.10876 (11)	0.2103 (2)	0.05401 (7)	0.0137 (3)	
C7	0.01049 (12)	0.4793 (2)	0.22402 (7)	0.0163 (3)	
H7A	−0.0450	0.5536	0.1862	0.020*	
H7B	0.0809	0.5363	0.2414	0.020*	
C8	0.10044 (12)	0.1762 (2)	0.25921 (7)	0.0182 (3)	
H8A	0.1687	0.2302	0.2707	0.022*	
H8B	0.0973	0.0438	0.2468	0.022*	
C9	−0.09404 (11)	0.1889 (2)	0.18263 (7)	0.0149 (3)	
H9A	−0.0970	0.0621	0.1660	0.018*	
H9B	−0.1552	0.2620	0.1474	0.018*	

### Atomic displacement parameters (Å^2^)

**Table:**

	*U*^11^	*U*^22^	*U*^33^	*U*^12^	*U*^13^	*U*^23^
O1	0.0162 (5)	0.0311 (6)	0.0120 (5)	0.0027 (5)	0.0085 (4)	0.0029 (4)
O2	0.0207 (5)	0.0237 (6)	0.0143 (5)	0.0014 (4)	0.0113 (5)	−0.0021 (4)
O3	0.0138 (5)	0.0258 (6)	0.0153 (5)	0.0029 (4)	0.0069 (4)	0.0023 (4)
O4	0.0198 (6)	0.0389 (7)	0.0128 (5)	−0.0009 (5)	0.0078 (5)	0.0008 (5)
O5	0.0306 (6)	0.0267 (6)	0.0222 (6)	0.0043 (5)	0.0194 (5)	−0.0029 (5)
O6	0.0256 (7)	0.0389 (9)	0.0517 (9)	−0.0181 (6)	−0.0076 (7)	0.0214 (7)
O7	0.0271 (7)	0.0164 (6)	0.0401 (8)	0.0023 (5)	−0.0020 (6)	0.0064 (5)
N1	0.0144 (6)	0.0126 (6)	0.0138 (6)	−0.0010 (5)	0.0083 (5)	0.0008 (4)
N2	0.0201 (6)	0.0165 (6)	0.0153 (6)	−0.0040 (5)	0.0114 (5)	−0.0029 (5)
N3	0.0138 (6)	0.0181 (6)	0.0164 (6)	0.0000 (5)	0.0096 (5)	0.0013 (5)
N4	0.0142 (6)	0.0167 (6)	0.0113 (5)	0.0000 (5)	0.0082 (5)	0.0000 (4)
C1	0.0150 (7)	0.0125 (7)	0.0137 (6)	−0.0023 (5)	0.0087 (6)	0.0003 (5)
C2	0.0138 (7)	0.0118 (6)	0.0147 (6)	−0.0013 (5)	0.0092 (6)	−0.0003 (5)
C3	0.0140 (6)	0.0108 (6)	0.0137 (6)	−0.0028 (5)	0.0073 (6)	−0.0002 (5)
C4	0.0192 (7)	0.0118 (7)	0.0133 (6)	−0.0034 (5)	0.0103 (6)	−0.0018 (5)
C5	0.0168 (7)	0.0105 (7)	0.0182 (7)	−0.0008 (5)	0.0120 (6)	−0.0006 (5)
C6	0.0129 (6)	0.0118 (6)	0.0156 (7)	−0.0006 (5)	0.0078 (6)	0.0024 (5)
C7	0.0199 (7)	0.0155 (7)	0.0142 (7)	−0.0031 (6)	0.0102 (6)	−0.0015 (5)
C8	0.0142 (7)	0.0256 (8)	0.0141 (7)	0.0063 (6)	0.0077 (6)	0.0023 (6)
C9	0.0137 (6)	0.0167 (7)	0.0127 (6)	−0.0030 (5)	0.0067 (6)	−0.0027 (5)

### Geometric parameters (Å, °)

**Table:**

O1—C1	1.2543 (17)	C2—C3	1.3876 (19)
O2—N1	1.2348 (16)	C3—C4	1.375 (2)
O3—N1	1.2297 (16)	C3—H3A	0.9500
O4—N2	1.2274 (17)	C4—C5	1.405 (2)
O5—N2	1.2307 (17)	C5—C6	1.361 (2)
O6—N3	1.2054 (19)	C5—H5A	0.9500
O7—N3	1.2037 (18)	C7—C7^i^	1.528 (3)
N1—C2	1.4535 (18)	C7—H7A	0.9900
N2—C4	1.4510 (18)	C7—H7B	0.9900
N3—C6	1.4718 (18)	C8—C9^i^	1.536 (2)
N4—C7	1.4930 (19)	C8—H8A	0.9900
N4—C9	1.4942 (18)	C8—H8B	0.9900
N4—C8	1.4952 (18)	C9—C8^i^	1.536 (2)
N4—H4A	0.9300	C9—H9A	0.9900
C1—C6	1.440 (2)	C9—H9B	0.9900
C1—C2	1.4409 (19)		
			
O3—N1—O2	122.45 (12)	C5—C4—N2	119.00 (13)
O3—N1—C2	118.70 (12)	C6—C5—C4	116.45 (13)
O2—N1—C2	118.83 (12)	C6—C5—H5A	121.8
O4—N2—O5	123.34 (12)	C4—C5—H5A	121.8
O4—N2—C4	118.88 (12)	C5—C6—C1	126.76 (13)
O5—N2—C4	117.78 (12)	C5—C6—N3	119.86 (13)
O7—N3—O6	123.01 (14)	C1—C6—N3	113.38 (12)
O7—N3—C6	118.46 (12)	N4—C7—C7^i^	108.68 (7)
O6—N3—C6	118.41 (13)	N4—C7—H7A	110.0
C7—N4—C9	110.72 (11)	C7^i^—C7—H7A	110.0
C7—N4—C8	109.79 (11)	N4—C7—H7B	110.0
C9—N4—C8	109.80 (12)	C7^i^—C7—H7B	110.0
C7—N4—H4A	108.8	H7A—C7—H7B	108.3
C9—N4—H4A	108.8	N4—C8—C9^i^	108.41 (11)
C8—N4—H4A	108.8	N4—C8—H8A	110.0
O1—C1—C6	119.29 (13)	C9^i^—C8—H8A	110.0
O1—C1—C2	128.51 (14)	N4—C8—H8B	110.0
C6—C1—C2	112.20 (12)	C9^i^—C8—H8B	110.0
C3—C2—C1	122.68 (13)	H8A—C8—H8B	108.4
C3—C2—N1	116.72 (12)	N4—C9—C8^i^	108.72 (11)
C1—C2—N1	120.56 (12)	N4—C9—H9A	109.9
C4—C3—C2	119.91 (13)	C8^i^—C9—H9A	109.9
C4—C3—H3A	120.0	N4—C9—H9B	109.9
C2—C3—H3A	120.0	C8^i^—C9—H9B	109.9
C3—C4—C5	121.92 (13)	H9A—C9—H9B	108.3
C3—C4—N2	119.08 (13)		
			
O1—C1—C2—C3	−178.37 (14)	N2—C4—C5—C6	179.14 (13)
C6—C1—C2—C3	1.4 (2)	C4—C5—C6—C1	2.8 (2)
O1—C1—C2—N1	4.2 (2)	C4—C5—C6—N3	−177.06 (12)
C6—C1—C2—N1	−175.98 (12)	O1—C1—C6—C5	176.35 (14)
O3—N1—C2—C3	10.33 (19)	C2—C1—C6—C5	−3.5 (2)
O2—N1—C2—C3	−167.99 (13)	O1—C1—C6—N3	−3.80 (19)
O3—N1—C2—C1	−172.10 (13)	C2—C1—C6—N3	176.37 (12)
O2—N1—C2—C1	9.59 (19)	O7—N3—C6—C5	−90.91 (18)
C1—C2—C3—C4	1.0 (2)	O6—N3—C6—C5	93.1 (2)
N1—C2—C3—C4	178.54 (12)	O7—N3—C6—C1	89.23 (18)
C2—C3—C4—C5	−1.9 (2)	O6—N3—C6—C1	−86.80 (19)
C2—C3—C4—N2	179.07 (13)	C9—N4—C7—C7^i^	55.03 (18)
O4—N2—C4—C3	5.2 (2)	C8—N4—C7—C7^i^	−66.36 (18)
O5—N2—C4—C3	−175.42 (13)	C7—N4—C8—C9^i^	56.78 (15)
O4—N2—C4—C5	−173.87 (13)	C9—N4—C8—C9^i^	−65.17 (13)
O5—N2—C4—C5	5.5 (2)	C7—N4—C9—C8^i^	−64.33 (15)
C3—C4—C5—C6	0.1 (2)	C8—N4—C9—C8^i^	57.06 (14)

Symmetry codes: (i) −*x*, *y*, −*z*+1/2.

### Hydrogen-bond geometry (Å, °)

**Table:**

*D*—H···*A*	*D*—H	H···*A*	*D*···*A*	*D*—H···*A*
N4—H4A···O1	0.93	1.69	2.589 (2)	161
N4—H4A···O2	0.93	2.42	2.954 (2)	117

## References

[bb1] Chen, L.-Z., Zhao, H., Ge, J.-Z., Xiong, R.-G. & Hu, H.-W. (2009). *Cryst. Growth Des.* **9**, 3828–3831.

[bb2] Dabros, M., Emery, P.-R. & Thalladi, V.-R. (2007). *Angew. Chem. Int. Ed.* **46**, 4132–4135. 10.1002/anie.200604830 17394267

[bb3] Ferguson, G. (1999). *PRPKAPPA* University of Guelph, Canada.

[bb4] Glidewell, C., Ferguson, G., Gregson, R. M. & Lough, A. J. (1999). *Acta Cryst.* C**55**, 2133–2136.

[bb5] Horiuchi, S., Ishii, F., Kumai, R., Okimoto, Y., Tachibana, H., Nagaosa, N. & Tokura, Y. (2005). *Nat. Mater.* **4**, 163–166. 10.1038/nmat1298 15665837

[bb6] Jin, Z.-M., Lin, C.-S., Wang, H.-B., Hu, M.-L., Shen, L. & Huang, L.-R. (2004). *Acta Cryst.* C**60**, o765–o767. 10.1107/S0108270104021936 15467153

[bb7] Kumai, R., Horiuchi, S., Sagayama, H., Arima, T.-H., Watanabe, M., Noda, Y. & Tokura, Y. (2007). *J. Am. Chem. Soc.* **129**, 12920–12921. 10.1021/ja075406r 17924632

[bb8] Rigaku (2005). *CrystalClear* Version 1.4.0. Rigaku Corporation, Tokyo,Japan.

[bb9] Sheldrick, G. M. (2008). *Acta Cryst.* A**64**, 112–122. 10.1107/S0108767307043930 18156677

